# Supplementary material for: Iterative improvement in the automatic modular design of robot swarms
Source: PeerJ Comput Sci. 2020 Dec 7;6:e322. doi: 10.7717/peerj-cs.322 (PMC7924708; doi:10.7717/peerj-cs.322)
Supplement: Supplemental Information 3 [file peerj-cs-06-322-s003.zip › argos3/doc/api/standalone/a00477_source.html]

ARGoS: plugins/robots/foot-bot/simulator/footbot\_turret\_encoder\_default\_sensor.h Source File


- Main Page
- Related Pages
- Namespaces
- Classes
- Files

- File List
- File Members

# plugins/robots/foot-bot/simulator/footbot\_turret\_encoder\_default\_sensor.h

Go to the documentation of this file.

```
00001 
00007 #ifndef FOOTBOT_TURRET_ENCODER_DEFAULT_SENSOR_H
00008 #define FOOTBOT_TURRET_ENCODER_DEFAULT_SENSOR_H
00009 
00010 #include <string>
00011 #include <map>
00012 
00013 namespace argos {
00014    class CFootBotTurretEncoderDefaultSensor;
00015 }
00016 
00017 #include <argos3/plugins/robots/foot-bot/control_interface/ci_footbot_turret_encoder_sensor.h>
00018 #include <argos3/plugins/robots/foot-bot/simulator/footbot_turret_encoder_default_sensor.h>
00019 #include <argos3/plugins/robots/foot-bot/simulator/footbot_turret_entity.h>
00020 #include <argos3/core/simulator/sensor.h>
00021 
00022 namespace argos {
00023 
00024    class CFootBotTurretEncoderDefaultSensor : public CCI_FootBotTurretEncoderSensor,
00025                                               public CSimulatedSensor {
00026 
00027    public:
00028 
00029       CFootBotTurretEncoderDefaultSensor();
00030 
00031       virtual ~CFootBotTurretEncoderDefaultSensor() {}
00032 
00033       virtual void SetRobot(CComposableEntity& c_entity);
00034 
00035       virtual void Update();
00036 
00037       virtual void Reset();
00038 
00039    private:
00040 
00041       CFootBotTurretEntity* m_pcTurretEntity;
00042 
00043    };
00044 
00045 }
00046 
00047 #endif
```

---

Generated on 10 Jul 2018 for ARGoS by 
 1.6.1
